# Managing undocked pigs – on-farm prevention of tail biting and attitudes towards tail biting and docking

**DOI:** 10.1186/s40813-016-0020-7

**Published:** 2016-02-01

**Authors:** Anna Valros, Camilla Munsterhjelm, Laura Hänninen, Tiina Kauppinen, Mari Heinonen

**Affiliations:** 1 Research Centre for Animal Welfare, Department of Production Animal Medicine, Faculty of Veterinary Medicine, University of Helsinki, PO Box 57, 00014 Helsinki, Finland; 2 Finnish Centre for Animal Welfare, University of Helsinki, PO Box 57, 00014 Helsinki, Finland

**Keywords:** Pig, Tail biting, Tail docking, Farmer attitudes, Risk factors

## Abstract

**Background:**

Tail biting is a common and serious welfare problem in pig production, causing large economical losses. Tail docking is performed routinely in most EU countries to reduce the tail biting risk. However, tail docking is painful, and does not prevent tail biting totally. The risk factors behind tail docking are multifactorial and most analyses are based on studies using biological or epidemiological approaches. There is very little information available on how producers deal with tail biting on-farm. There are also no studies on the attitude of producers towards tail docking and tail biting in systems with long-tailed pigs. We aimed to study how farmers rate the efficiency of different measures for preventing and intervening with tail biting, when tail docking is not allowed. Furthermore, we investigated the attitudes of Finnish farmers to tail docking and tail biting.

**Results:**

Respondents scored feeding-related issues to be most important for prevention of tail biting, identifying and removing the biting pig as most important intervention measures, and straw as the most important manipulable material when preventing tail biting. Tail biting was not perceived as a very serious problem by over 70 % of the respondents, even though docking is not allowed, and was reported to occur close to a level which was also considered acceptable by the respondents. Most respondents did not think it is probable they would raise tail docked pigs if it were possible, but about 21 % probably would.

**Conclusions:**

In comparison with other authors’ findings, the ranking of importance of risk factors for tail biting differs between scientists and farmers, and between farmers in different cultures of pig production. In addition, the attitude towards tail biting and tail docking appears to be very different in producers with different experiences of tail docking. These results indicate that a scientist-farmer dialogue, as well as international communication is important when trying to reduce the risk of tail biting, and subsequently the need for tail docking.

## Background

Tail biting is a common and serious welfare problem in pig production. Tail biting has been connected to increased stress level of the pigs [1] and risk factors include a wide range of housing- and management-related problems, as well as characteristics of individual pigs [2]. Bracke et al. [3] found that Dutch producers thought ‘biting’ was one of the main welfare problems in pig production. Tail biting also causes considerable economical losses [4, 5].

Tail docking is a painful procedure [6, 7] performed to reduce the risk of tail biting. By docking the tail, the tail biting risk decreases, although the procedure does not eliminate tail biting: in a recent study in Ireland, where 99 % of the pigs are docked, a prevalence of tail lesions of 72.5 % (including mild lesions) was still reported at the abattoir, with 2.5 % severe lesions [5]. Based on available studies, Valros & Heinonen [8] suggested that tail docking reduces the prevalence of severe lesions by about 50 %. Tail docking in itself, in addition to causing acute, and possibly chronic pain [9], reduces the risk of tail biting when keeping pigs in rearing systems with a higher level of risk factors, and thus might increase the risk of keeping pigs in a way that is not optimal for their welfare [8, 10]. Another important reason for favouring long-tailed pigs is that in non-docked pigs, the status of the tails can be used as a measure of the overall welfare status on the farm [11]. As it is reasonable to expect that tail biting will increase as a result of not docking, at least in the initial stage [3], it is also important to gain knowledge about what would be an acceptable rate of tail biting considering animal welfare implications, farmers’ views and societal considerations [12].

The multifactorial nature of the tail biting problem is illustrated by the on-farm intervention tool HAT developed in the UK [13]: it includes 83 risk factors, divided into eight categories. Thus, there is no simple measure for reducing tail biting that applies to all farms. It has been suggested that managing the risk factors behind tail biting needs to be considered on a farm-specific level [2]. The majority of experimental studies on tail biting risk factors have been on manipulable materials (for a review, see [12]), while risk factors related to e.g. feeding [14, 15], climate issues [16, 17] and reduced health [14, 18, 19] have been identified in epidemiological studies.

It has been shown that the view of how to prevent tail biting differs between producers and scientists. A study, including Dutch farmers and scientists, showed that producers did not agree with the scientists' focus on ‘boredom’ being the most important reason for tail biting [20]. Also Bracke et al. [3] suggested differing views on tail biting and docking between producers and scientists. Bernard et al. [20] indicated that farmers might ignore scientific information, maybe because it is not concrete enough, or too focused on specific factors. The authors suggest that farmers have a more holistic approach, while scientists tend to reduce reality into more controllable facts [20]. This indicates that there is a need to listen to farmers in order to fully understand the problems related to tail biting, and in order to enhance communication between science and end-users. There are, however, very few studies asking farmers about their view on how to most efficiently prevent tail biting. Bracke et al. [3] studied attitudes of Dutch farmers towards tail biting and docking, as well as their views of efficiency of preventive and intervention measures. The Dutch producers rated climatic factors as most important, and conventional producers agreed to a large extent with the statement that it is better to dock all pigs than risk any cases of tail biting. Hunter et al. [15] asked farmers in the UK about which intervention measures they mostly use and found that removal of the bitten pig or the biter, and adding objects for manipulation were the most commonly used measures. However, to our knowledge there is no comparable data from conventional farmers not docking their pigs, while a need for such information has been identified [10].

Attitudes do not only differ between different stakeholder groups, but producer attitudes to animal welfare appear to differ between countries. For example, in a study of Canadian pig producers, the producers did not really feel comfortable using the term animal welfare at all [21], while Finnish producers do not seem to find this a problem, even though their definition of animal welfare might differ from that commonly used by scientists [22]. The producers in the study by Kauppinen et al. [22] most often mentioned animal welfare in the concept of providing the animals with a favourable living environment and good healthcare. As the tail biting issue is closely related to animal welfare, cultural and societal differences between countries might also affect attitudes towards tail biting and docking. Finland is an interesting country in this context, as tail docking has never been widely applied, and as it has been totally banned since 2003. Views might be influenced by the system farmers are used to [21], thus it can be expected that the view of Finnish farmers might differ from e.g. that of the Dutch producers in the study by Bracke et al. [3].

We aimed to study how farmers rate the efficiency of different measures for preventing tail biting, when tail docking is not allowed. In addition, we studied which tail biting intervention strategies farmers find most efficient, and which manipulable materials are used, and how efficient these are found to be in preventing tail biting. The preventive and intervention measures included in the study were selected based on existing scientific information. Furthermore, we investigated the attitudes of Finnish farmers to tail docking and tail biting.

## Results

In total, we received 70 replies, which represents about 7.5 % of Finnish pig farms. Of these, 44 were from fattening farms, 14 from piglet producing farms, and 12 from integrated farms (farrow to slaughter). Each response represented either a fattening unit (54) or a weaning unit (16). The respondents had an average experience of 18 years within pig farming (median 18, range 1–44 years), and the average unit size (size of the unit the response was representing) was 1307 pigs (median 875, range 100–6400 pigs). The average unit size of the respondents is thus slightly larger than that of the estimated average Finnish farm. The division of farms between different farms size classes is presented in Table 1.

**Table 1:** Number of farms within different farm size categories

Number of pigs^a^	Number of farms	% of respondents
<500	25	35.7
500–1000	16	22.9
1000–2000	19	27.1
>2000	10	14.3

^a^Number of animals within the production phase which the questionnaire answers represent

### Perceived importance of preventive measures

All the measures included in the questionnaire were perceived as being at least somewhat important, as they all scored over 4 on average. ‘Enough feeding space’ was the top-rated measure, also showing a low variation between respondents, closely followed by ‘Taking care of animal health’. Also ‘Managing air movements’, ‘Water available to all pigs’, ‘Correct feed content’ and ‘Good quality pigs’ got average scores of 6 or above (Table 2). As a whole, the measure subcategory ‘Feed and water’ scored significantly higher (median: 6.25 (interquartile range: 1.50)) than all the other subcategories (*p* < 0.001 for all comparisons). The average score of the subcategory ‘Housing and environment’ (5.14 (1.43)) was significantly lower than all the other subcategories (*p* < 0.001 for all comparisons). The subcategories ‘Pig behaviour’ (5.60 (1.20)) and ‘Animals’ (5.50 (1.13)) scored intermediate.

**Table 2:** Perceived importance of the different preventive measures given in the questionnaire

Measure	Subcategory^e^	Average (standard deviation)^f^	Median (interquartile range)^f^
Enough feeding space^a^	PB	6.30 (0.92)	7 (1)
Taking care of animal health	A	6.21 (0.95)	7 (1)
Managing air movements (draught)	HM	6.16 (1.16)	6 (1)
Water available to all pigs	FW	6.10 (1.36)	7 (2)
Correct feed content	FW	6.10 (0.98)	6 (2)
Good quality pigs (healthy, evenly grown)	A	6.00 (1.16)	6 (2)
Even quality of feed	FW	5.96 (0.94)	6 (2)
Managing air quality	HM	5.81 (1.27)	6 (2)
Appropriate temperature in pen	HM	5.71 (1.26)	6 (2)
Feeding always at the same time	FW	5.71 (1.53)	6 (2)
Restricting animal density	PB	5.66 (1.42)	6 (2)
Background of the piglets^b^	A	5.53 (1.40)	6 (2)
Use of bedding-type materials^c^	PB	5.46 (1.62)	6 (2)
Avoiding mixing of animals	PB	5.06 (1.61)	5 (3)
Managing noise level	HM	4.79 (1.54)	5 (2)
Breed of pigs	A	4.77 (1.75)	5 (2)
Use of objects for manipulation^d^	PB	4.61 (1.79)	5 (3)
Adjusting natural light from windows	HM	4.49 (1.70)	5 (3)
Adequate light level	HM	4.27 (1.46)	4 (1)
Managing pen hygiene/cleanliness	HM	4.21 (2.05)	4 (3)

^a^Enough feeding space for each pig at the trough or enough feeding places at the feeding automat

^b^Such as housing and management in farrowing or weaning unit

^c^Such as straw, wood-shavings/sawdust, peat

^d^Such as chains, wood

^e^Measure subcategories: *HM* housing and environment, *PB* pig behaviour, *FW* feed and water, *A* animals

^f^Scale: 1 (not important at all) to 7 (very important)

Also in the open answers on further measures the producers had found important, feeding-related measures were mentioned most frequently (by 17 respondents). The need to feed over the recommended norms, not to feed more than the pigs can eat, to have enough feeding times and to give enough minerals and salts were mentioned, as well as the importance of correct feed composition. The importance of outside conditions were mentioned in seven replies: noise and light from the outside are problematic, as well as variation in outside temperature. Six respondents mentioned the importance of the caretaker: the time spent by the caretaker in the piggery (which was mentioned both as a protective and as a risk factor), as well as the risk of changing caretaker. Further open answers included the importance of variation in manipulable materials, the background and quality (such as tail damage status) of pigs on arrival and keeping pig groups stable.

### Intervention measures

When tail biting had already started in a pen, identification and removing the biter were reported as the most important intervention measures. These were rated significantly higher than all the other measures (*p* < 0.05 for all comparisons). ‘Adding bedding-type material’ (*p* < 0.001 for all comparisons), and ‘Removing the bitten pig from the pen’ (*p* < 0.05 for all comparisons) were considered significantly more important than ‘Adding objects for manipulation’, ‘Reducing animal density’ and ‘Use of anti-biting substances on the tail’ (Table 3).

**Table 3:** Perceived importance of the different tail biting intervention measures given in the questionnaire

Measure	Average (standard deviation)^5^	Median (interquartile range)^5,6^
Identifying the biter	6.49 (0.97)	7 (1)^a^
Removing the biter from the pen	6.29 (1.08)	7 (1)^a^
Adding bedding-type material^1^	5.79 (1.34)	6 (2)^b^
Removing the bitten pig from the pen	5.39 (1.30)	6 (1)^b^
Adding objects for manipulation^2^	4.71 (1.62)	5 (2)^c^
Reducing animal density^3^	4.60 (1.70)	5 (2)^c^
Use of anti-biting substances on the tail^4^	4.31 (1.10)	5 (3)^c^

^1^Such as straw, wood-shavings/sawdust, peat

^2^Such as chains, wood

^3^By removing (any) pigs from the pen

^4^Such as tar, Porcivet®

^5^Scale: 1 (not important at all) to 7 (very important)

^6^Different subscripts indicate differences in median rating between the factors (*p* < 0.05)

Also regarding intervention measures, feeding-related factors were mentioned most often in the open answers (by 14 respondents). Most often (9 respondents) the suggestion was to add minerals, salts or other additional feedstuffs, mainly on the floor of the pen. Regarding addition of manipulable materials the respondents underlined the importance of adding something novel and variable to the pigs. A few respondents also mentioned the importance of making sure the victim’s bitten tail heals quickly, by using e.g. wound spray and medication, to avoid further interest in the tail.

### Manipulable materials used for prevention of tail biting

None of the manipulable materials named in the questionnaire got an average score over 6, and all of them divided the opinion of the respondents to a higher degree than most of the preventive and intervention measures described above. Straw, newspaper, hay and cardboard or paper sacks scored on average over 5. In general, bedding type materials scored higher than objects. Straw, newspaper and chains were the most commonly used materials (Table 4).

**Table 4:** Perceived efficiency in preventing tail biting of the different manipulable materials given in the questionnaire

Material	*N* ^c^	Average (standard deviation)^d^	Median (interquartile range)^d^
Straw	61	5.64 (1.74)	6 (2)
Newspaper	61	5.44 (1.55)	6 (3)
Hay	37	5.21 (1.82)	6 (3)
Cardboard or paper sacks	46	5.13 (1.58)	5 (2)
Chain	58	4.75 (1.79)	5 (3)
Unprocessed, fresh wood^a^	47	4.63 (1.81)	5 (3)
Sawdust/wood shavings	52	4.57 (1.94)	5 (4)
Rope	45	4.41 (1.64)	5 (2)
Peat	29	4.37 (2.08)	4.5 (3)
Ball or other object attached to a chain	47	4.31 (1.78)	4 (3)
Processed, dry wood^b^	34	3.89 (1.91)	4 (4)
Ball or other object loose on the floor	53	3.71 (1.72)	4 (3)
Commercial pig toy	39	3.68 (1.51)	4 (2)

^a^Such as branches, a piece of tree trunk

^b^Such as a piece of plank

^c^Number of respondents that had experience of the material in question

^d^Scale: 1 (not at all efficient) to 7 (very efficient)

On average, the respondents reported adding 0.4 l of bedding material per pig per day (median: 0.3, range 0.02–1.5 l). One farm reported having deep bedding in the pens.

The respondents mentioned a few further manipulable materials in their open answers, but no materials came up very strongly. The mentioned materials included, among others, silage, used car tires, straw pellets, pieces of watering hose, stones, empty plastic containers, and used boots and shoes. Also here, the respondents underlined the importance of variation in manipulable materials, as well as that the materials need to be movable by the pigs.

### Producer attitudes towards tail biting and docking

The TB level was reported as on average 2.3 % (median 1 %, range 0–30 %). Tail biting was not considered a very serious problem: 72 % of the respondents replied to the question of how serious a problem tail biting was on their farm (TB problem) with score 1 or 2 (Table 5). Of the respondents, 29 % did not consider tail biting to be acceptable at all (TB acceptable). A level of 1–2 % tail biting was considered to still be acceptable by 51 % of the respondents, while 2.9 % thought that even 11–15 % is acceptable (Table 5).

**Table 5:** a-c The distribution of answers for the seriousness of the tail biting problem (5a); the probability of docking, if docking was legal (5b); and the level of tail biting found to be acceptable (5c)

5a Scale	TB problem^4^	5b Scale	TD probability^5^	5c % tail biting	TB acceptable^6^
1^2^	37.1 (26) ^1^	1^3^	34.3 (24)^1^	0	28.6 (20)^1^
2	34.3 (24)	2	25.7 (18)	1–2	50.0 (35)
3	12.9 (9)	3	4.3 (3)	3–4	14.3 (10)
4	8.6 (6)	4	7.1 (5)	5–7	2.9 (2)
5	2.9 (2)	5	7.1 (5)	8–10	1.4 (1)
6	2.9 (2)	6	5.7 (4)	11–15	2.9 (2)
7	1.4 (1)	7	15.7 (11)	>15	0 (0)

The information given in the rows do not represent the same herds

^1^Data is given as percentage and (*N*) of replies

^2^Scale: 1: not a problem at all–7: a very serious problem

^3^Scale: 1: definitely not–7: very probably

^4^How serious a problem do you perceive tail biting to be on your farm?

^5^Tail docking is forbidden in Finland. If it was allowed, how probable is it that you would raise docked pigs?

^6^How much tail biting do you perceive as acceptable?

The majority (62 %) of the respondents thought it was very unlikely that they would raise docked pigs even if it was made legal in Finland (score 1 or 2) (TD probability). On the other hand, about 21 % said they would probably (score 6 or 7) raise docked pigs if it were possible (Table 5).

Typically, respondents who stated they would most probably not tail dock even if it was legal, motivated this with ethical arguments, such as that the pig has the right to its tail, that tail docking is animal cruelty, or that it is inhumane to dock pig tails. They also referred to the pain induced by docking, and the increased risk for infections due to docking as well as to the importance of the tail as a welfare indicator. One of the respondents, a farmer with an integrated unit and 27 years of experience stated that:

“*[Tail docking] causes the pigs unnecessary pain and increased risk for infections – why should we dock when we can manage well with long-tailed pigs*”.

On the other hand, respondents that stated they would very probably raise tail docked pigs if it was legal, motivated their view with more utilitarian reasons, such as that docking increases farm profit, reduces work load and decreases medication need, thus resulting in cleaner meat. They did, however, also refer to tail docking making life easier for the pigs, and reducing the risk for joint infections in pigs. A fattening pig farmer, with 15 years of experience of raising pigs stated that:

“*[Tail docking] is the only right thing to do in efficient production. The Finns are giving other countries a huge competitive advantage*”.

### Correlations between different variables

There were several correlations between the attitude towards tail docking, perceived seriousness of tail biting, tail biting occurrence on the farm, level of perceived acceptable tail biting level, and farm size (Table 6). The correlation between TD probability and farm size disappeared if the smallest farm class (<500 pigs) were excluded from the analyses (*p* = 0.7). The number of years the respondents had experience of raising pigs did not correlate with any of these variables (*p* > 0.05 for all).

**Table 6:** Correlations between attitudes towards tail biting and docking, occurrence of tail biting and farm size

	TB problem	TB acceptable	TD probability	Farm size
How much tail biting occurs on your farm? (TB occurrence)	r^a^ = 0.43 *p* < 0.001	r = 0.41 *p* = 0.001	r = 0.31 *p* = 0.01	ns^b^
How serious a problem do you perceive tail biting to be on your farm? (TB problem)		ns	r = 0.34 *p* = 0.005	ns
How much tail biting do you perceive as acceptable? (TB acceptable)			r = 0.22 *p* = 0.07	r = 0.25 *p* = 0.04
Tail docking is forbidden in Finland. If it was allowed, how probable is it that you would raise docked pigs? (TD probability)				r = 0.31 *p* = 0.009

^a^Spearman rank correlations

^b^ *ns* correlation non-significant, *p* > 0.1

## Discussion

The farmers generally agreed with the high importance of measures to prevent tail biting that have been shown to be important also in experimental and epidemiological studies. The farmers did, however, underline the importance of feeding-related factors more than previous science has, while giving the importance of manipulable materials less weight. The respondents do, in general, not find tail biting a very large problem on their farms, despite not tail docking. The majority of the farmers would not want to raise docked pigs even if it was legal in Finland, even though there is a subset of farmers that indicate they would most probably do so if they could.

It is important to keep in mind that this study is based on an open questionnaire, with a rather small response rate, which makes it impossible to generalise the results to the entire Finnish pig farmer community. It is possible that the responses represents farmers most interested in the topic. However, the subset of farms the respondents represent, do appear to be rather representative of Finnish farms regarding tail biting incidence, and to represent a slightly larger farm size than average in Finland. As the vast majority (estimated by the authors to be at least 95 %) of pig farmers in Finland use computerized production recording systems, the fact that the questionnaire was performed on-line should not affect the study population significantly. It should also be noted that the main aim of the study was not to generalize nationally, but to investigate experiences and thoughts of farmers used to deal with long-tailed pigs.

The respondents in this study agreed with the general importance of the preventive measures listed in the questionnaire, with all measures getting a median score of four or more on a scale from one (not important at all) to seven (very important). This shows that there is a certain level of agreement between farmers and the existing science. However, the ranking of the different measures differed to a large extent from the ranking by experts presented in an EFSA opinion [23]. Experts in the EFSA opinion [23] ranked risk factors related to absence of manipulable material as clearly most important, followed by retarded growth within the group, and castration. Feeding and diet-related issues were valued as less important risk factors. This was the opposite in this questionnaire: the respondents ranked feeding related factors very high, especially competition for feed, while the addition of manipulable material was ranked lower than expected. Also animal health was ranked very high.

The fact that manipulable material was ranked lower than expected from expert opinions is in accordance with the results by the study by Bracke et al. [3], where pig farmers scored boredom of the pigs to be only the 7th most important factor in preventing tail biting. Farmers in the study by Bernard et al. [20] criticized scientists for focusing more or less solely on manipulable materials when trying to solve the tail biting problem. These researchers also showed that Dutch producers do not believe boredom being a reason for tail biting, and even think that domestic pigs have lost the need to root. Another reason for the respondents in this questionnaire to rank manipulable materials lower than expected might be that their view is influenced by the system the farmer is used to [21]. In Finland, some level of bedding-type manipulable material is compulsory. Thus it is possible that farmers take the use of manipulable material as self-evident, and thus do not see the effect on tail biting in practice. However, this strict definition of the regulation of use of manipulable material has been implemented only for a few years, so at least some of the farmers most probably also have experience of raising pigs without the use of bedding-type manipulable material. One reason for the main focus on manipulable materials by researchers might be due to the mere fact that the largest number of experimental and epidemiological studies on risk factors for tail biting have focused on this [12]. Farmers, on the other hand, might see the situation on their farm in a more holistic way [20], identifying also more subtle risk factors.

The Dutch farmers in the study by Bracke et al. [3] ranked ‘Stable climate’ as the most important factor, while the subcategory ‘Housing and Environment’ did not receive a very high scoring in the current study, nor according to expert opinion [23]. The fact that competition for feeding is an important tail biting risk is not a new finding: it has been reported in previous studies [14, 24]. Also animal health is an issue receiving more and more attention as a risk factor for tail biting [18], but so far, the link between tail biting and health is still inconclusive [12].

An interesting factor that came up in the open answers, and which has not received much attention previously, is the importance of the caretaker. This underlines the importance of experience of non-docking pigs, which might explain why farmers converting to non-docking might frequently encounter problems with tail biting [3]. This might also be an explanatory factor for why Finland can manage so well with the non-docking policy: Finnish farms are, on average smaller than in many other EU countries. On large farms, often with a reduced caretaker/pig ratio, it becomes more difficult to spot tail biting early enough, and have time to intervene appropriately [10]. However, we did not find a correlation between farm size and occurrence of tail biting in this study. Also the expert opinion reported by EFSA [23] did not consider herd size as a very important risk factor. Interestingly, even though tail biting is not related to farm size in this study, larger farms perceive tail biting as a more serious problem, and are more willing to dock. Also Bracke et al. [3] reported that very large farms were less motivated to stop docking than smaller ones.

There are very few studies on how to efficiently intervene when tail biting is about to start, or when it has already started. The only experimental study on these compared the effect of removing the biter to adding straw, and found these measures equal [25]. The study, however, did not include a control without intervention. The most commonly used intervention measure reported by producers in UK [15] was removing the bitten pig, followed by adding manipulable objects and removing the biter. Adding bedding-type materials was only used by a minority of the farmers, while this was ranked rather high by the respondents in the current study. Dutch farmers [3] reported removing biters and bitten animals from the group when tail biting occurs most frequently. Removing pigs, especially in the case of victims during outbreaks, might be a challenge, as enough sick pens on the farm are necessary. It is also not sure if biters can be combined into common groups without further tail biting problems. This might however not be a problem, as reported by Zonderland et al. [25], and is also mentioned by one respondent in the current study: “*It is worthwhile to combine biters into groups, they do not necessarily bite each other*”.

Teeth clipping of biter pigs, which was mentioned as a rather common procedure by the Dutch producers [3], and which is illegal in both the Netherlands and Finland was mentioned only by one respondent in the current questionnaire.

Edwards [26] mentioned adding salt to tail biting pens to stop tail biting. Adding salts, minerals or other supplementary feedstuffs to the pens when tail biting is about to start, or has started, was also a popular measure mentioned in the open answers of this study. There are some commercial feeds available to use for tail biting intervention, further indicating the potential efficiency of this measure. As far as we know there is, however, only one study investigating the effectiveness of only one such feed in reducing tail biting, and it failed to show an effect [24], indicating a need to study other feeds.

Using anti-biting substances was not ranked very high in this study, which is in agreement with the low level (by less than 5 % of farmers) of use by Dutch farmers [3]. About 25 % of the UK farmers in the study by Hunter et al. [15], however, reported using such substances. Bracke et al. [27] showed experimentally that ropes can be made less attractive to pigs by adding tar.

The ranking of efficiency of manipulable materials in this study, with bedding-type materials scoring best, correspond rather well with previous reports, such as the study by Bracke et al. [28] comparing the outcomes of 54 different materials. The ranking also corresponds to the finding that pigs prefer materials that are complex, ingestible, odorous, chewable, deformable and destructible and contain sparsely distributed edible parts [29, 30]. Similar criteria were also considered important by experts in the study by Bracke et al. [31]. Slightly surprisingly, chains were ranked relatively high in the current study, even though they do not meet the before-mentioned criteria very well, and have been suggested not to be sufficient to meet the need for manipulation in pigs [32]. However, chain was one of the most commonly used of the manipulable materials, only straw and newspaper was used by a few more of the respondents. It might thus be that the high ranking is partly due to the producers being used to using chains, and this influences their response. Also in the Netherlands, farmers use chains very frequently (52–63 %), while straw was used by only 2.1–3.1 % of the farmers [3]. Even though Finnish farmers seldom use straw bedding, and as reported in this study, rather moderate amounts of bedding-type material, the regular addition of this type of material might be crucial for raising long-tailed pigs successfully.

The attitude towards tail docking in the current study was divided: the majority did not favour tail docking, but a considerable proportion (21 %) of the respondents would prefer to raise docked pigs if it were possible. This polarization of opinions might partly be due to the questionnaire being totally voluntary and open to anyone interested, which might cause people with strong opinions to be more inclined to answer. The different view on tail docking compared to the study by Bracke et al. [3], where farmers scored on average 4.9 on the statement ‘Docking is necessary to prevent tail biting’, on a scale from one (completely disagree) to six (completely agree) might be explained by the farmers defending what they currently do in both countries [21]. It might also be that this in the case of the Dutch farmers, who are used to docking tails, the efficiency of tail docking might be slightly overrated, as recent studies indicate that tail biting is a major problem also in docked pigs [5]. However, the Dutch farmers that reported having tried to stop tail docking did often encounter problems [3], which might strengthen this opinion even further. It needs to be remembered that the Finnish producers are used to not docking.

The actual level of tail biting on the farm is related to the attitudes of the farmer: the higher the reported tail biting, the higher the perceived seriousness of the problem, and the higher the probability of wanting to dock if it was allowed. As we base our data on tail biting occurrence on the responses in the questionnaire, and not on more objective data, we cannot, however exclude the fact that the link here might not be the opposite: the more serious a problem the farmer perceives tail biting to be, the more he/she focuses on it, and thus perceives there to be more tail biting on his/her farm than a farmer perceiving the problem as less serious.

While the Dutch farmers agreed very much with the statement that ‘It is better to dock all tails than to run the risk of tail biting even if it concerns just one bitten pig’ (Score 5 on the scale mentioned above), the respondents in the current study seem to think it is better to accept some tail biting than to dock all pigs. The majority of the respondents think a certain amount of tail biting is acceptable, while the vast majority do not think tail biting is a serious problem on their farm. Again, this is very different from Dutch farmers, which, despite raising tail-docked pigs, think biting is the main welfare problem on farms [3].

The motivations for how probably the respondent would raise docked pigs if it was legal were very different depending on the score given: Respondents who scored the probability of raising tail docked pigs high motivated this by practical, utilitarian reasons. On the other hand, scoring the probability of wanting to dock low was frequently motivated by reasons such as the pig having a right to its tail, and by docking being cruel. On the contrary, conventional Dutch pig farmers seemed to mainly disagree with the statement that a curly tail is important for sustainable pig husbandry (2.7 on a scale one (completely disagree) to six (completely agree)) [3]. Interestingly, similar motivations both for docking, and against docking, were also used, one example being that an increased infection risk was mentioned both due to docking, and due to not docking (as a consequence of increased tail biting).

The tail biting level (average 2.3 %, median 1 %) on the farms in this is close to what has been reported by Finnish slaughterhouses (2.3 % in 2013, [8]), using the same definition of severity. It needs to be noted however, that this, by definition, only includes severe, clinical cases, and total tail lesion level (including milder lesions) is probably much higher, as reported recently by Harley et al. 2014 [5] and in a study of Finnish pigs in 2000 [33]. This also corresponds rather well with the level considered acceptable by the majority of the respondents (50 % said that 1–2 % is manageable), supporting the fact that the respondents did not perceive tail biting as a very big problem on their farms. The more tail biting occurred on the farm, the higher the limit for what was thought to be acceptable was; showing that farmers are used to what they have now.

The high agreement by Dutch farmers with the statement ‘It is better to dock all tails than to run the risk of tail biting even if it concerns just one bitten pig’ in the study by Bracke et al. [3] indicates that they are very dependent on tail docking as a measure of preventing tail biting. As long as this is the case, it will be very difficult to implement a total ban on tail docking. Instead, it is important to communicate that farmers should not expect tail biting to disappear before tail docking can be banned [8], but that there is a need to find a level with which the producers can manage. This acceptance of a certain level of tail biting might be another factor why Finnish farmers manage with the tail docking ban. Even though 90 % of the respondents in this study reported some level of tail biting on their farm, the majority did not think this was a serious problem. In the study by Bracke et al. [3], only 35–50 % of the farmers reported tail biting to occur on their farms, and at a level of 1–5 %. In the study by Bracke et al. [3], tail biting was not defined in detail, but as ‘tail wounds’.

This study further shows that there is a need to realise that farmers might not always agree with, or accept scientific results, as also suggested by Bernard et al. [20]. Farmers might not observe as scientists, but they are present on their farms seven days a week, and might have a more holistic approach, instead of focusing on details [20]. However, a high level of production has been connected to a positive attitude towards researchers as a source of knowledge in piglet producing farms [34]. There might thus be a connection between a professional attitude towards pig production and an interest in scientific knowledge, which indicates the importance for a dialogue between scientists and producers.

As suggested in the EFSA opinion from 2014 [2], tail biting prevention needs to be considered on an individual farm level. Taylor et al. [13] showed that when applying the HAT tool on-farm there is a big variation between farms with similar total scores in how the different subcategories are scored. They also showed that reasonably low risk scores can be achieved in different types of systems, e.g. with and without straw bedding, and that risk scores can be reduced successfully on existing farms without major changes of the housing system. The importance of individually tailored tail biting prevention for different farms is also indicated by farmers in our questionnaire suggesting opposite measures for reducing the risk of tail biting in some cases: for example, some underlined the importance of feeding enough, even over the recommended norm, while one farmer clearly stated the feeding too much is a risk factor. Another example was related to the caretaker: some farmers said you need to spend lots of time with the pigs, while others indicated the importance of not being in the piggery more than necessary. However, an alternative explanation for these contradictive statements is that farmers might not always know themselves, which measures actually work on their farm.

## Conclusions

Respondents scored feeding-related issues to be most important for prevention of tail biting, identifying and removing the biting pig as most important intervention measures, and straw as the most important manipulable material when preventing tail biting. Tail biting was not perceived as a very serious problem, even though docking is not allowed, and was reported to occur close to a level which was also considered acceptable by the respondents. Most respondents did not think it is probable they would raise tail docked pigs if it were possible, but about 21 % probably would. In comparison with other authors’ findings, the ranking of importance of risk factors for tail biting differs between scientists and farmers, and between farmers in different cultures of pig production. In addition, the attitude towards tail biting and tail docking appears to be very different in producers with different experiences of tail docking. These results indicate that a scientist-farmer dialogue, as well as international communication is important when trying to reduce the risk of tail biting, and subsequently the need for tail docking.

## Methods

An online questionnaire was distributed in the spring of 2015 via producer-aimed webpages of the three biggest slaughterhouses in Finland, as well as via the web-pages of the Finnish Pig Entrepreneur Association (Suomen Sikayrittäjät ry) and several social media. The questionnaire was also advertised via the national producers’ newspaper (Maaseudun tulevaisuus). The questionnaire was open for responses for 1 month (May 18th to June 19th 2015).

Due to the open method of data collection, the questionnaire was theoretically available to the entire Finnish pig producer community. In total, there were 932 farms with pig production as their main source of income in Finland in 2014 [35]. These farms, which represent all farm types and production phases, had on average 1336 pigs per farm. This number is higher than the estimate for the size of fattening pig farms in Finland by D’Eath et al. [12], which was 485 animals on average in 2010. This discrepancy is due to the former number including piglet producing farms, with all production phases, as well as partly due to a continuing increase in farm size.

The questionnaire was designed to be as short, and as easy as possible for the producers to fill in. It included a few general questions (farm type and size of unit, farmer experience), and respondents were asked to reply concerning one production phase only per questionnaire (weaning pigs, fattening pigs or breeding gilts). The questionnaire further included sets of questions to evaluate the perceived importance of preventive measures (Table 2) and intervention measures (Table 3), using a scale from 1 (not important at all) to 7 (very important). The preventive measures were divided into four subcategories: Housing and environment; Pig behaviour; Feed and water; Animals. Furthermore, farmers were asked to evaluate the efficiency of different manipulable materials in preventing tail biting (Table 4) on the scale 1 (not at all efficient) to 7 (very efficient), and to estimate how much bedding-type material they use per pig per day. Producers were also given the opportunity to write open comments and suggestions on all the above topics.

In the last part of the questionnaire, producers were asked how serious a problem they perceive tail biting to be on their farm (1: not a serious problem at all–7: a very serious problem) (TB problem), and how much tail biting they find to still be acceptable (TB acceptable). Finally, we asked how probably the farmers would raise tail-docked pigs if it was allowed in Finland (TD probability) (1: not at all probable–7: very probable), and asked them to motivate their answer. Respondents were also asked about the level of tail biting on their farm (TB level), either by using feedback from the slaughterhouse or health care registrations, or estimated by themselves. The respondents were told to define tail biting as a tail with clear biting marks and / or blood, or the tail is clearly shortened due to biting.

### Statistical analyses

All statistical analysed were performed with IBM SPSS statistics 21.

Descriptive data is given both as median and interquartile range and as average and standard deviation. All statistical tests are performed using non-parametric tests, as most variables were non-normally distributed, and as the majority of the variables are non-continuous. Because the median is rather invariable, and thus does not discriminate differences very well in data based on a categorical scale, the perceived efficiency of the different measures to prevent tail biting and intervention measures are ranked using average values.

The difference in the average score for the prevention measure subcategories, as well as the difference in perceived importance of intervention measures was tested using Friedman’s two-way analyses followed by Wilcoxon rank sum test for pairwise comparisons when appropriate.

Connections between perceived seriousness of the problem of tail biting, occurrence of tail biting on the farm, and probability that the respondent would raise docked pigs if it was legal were tested with Spearman rank correlations, as was the effect of unit size and experience of the respondents in raising pigs on these variables.
